# Phylogenetic determinants of toxin gene distribution in genomes of *Brevibacillus laterosporus*

**DOI:** 10.1016/j.ygeno.2019.06.020

**Published:** 2020-01

**Authors:** Travis R. Glare, Abigail Durrant, Colin Berry, Leopoldo Palma, M. Marsha Ormskirk, Murray P. Cox

**Affiliations:** a Bio-Protection Research Centre, PO Box 85084, Lincoln University, Lincoln, New Zealand; b Cardiff School of Biosciences, Cardiff University, Museum Avenue, Cardiff CF10 3AX, UK; c Universidad Nacional de Villa María, Instituto A.P. de Ciencias Básicas y Aplicadas, Av. Arturo Jauretche 1555, Villa María 5900, Córdoba, Argentina; d Statistics and Bioinformatics Group, Institute of Fundamental Sciences, Massey University, Palmerston North 4410, New Zealand

**Keywords:** Genomes, Toxins, Phylogeny, Virulence, Bacillales

## Abstract

*Brevibacillus laterosporus* is a globally ubiquitous, spore forming bacterium, strains of which have shown toxic activity against invertebrates and microbes and several have been patented due to their commercial potential. Relatively little is known about this bacterium. Here, we examined the genomes of six published and five newly determined genomes of *B. laterosporus,* with an emphasis on the relationships between known and putative toxin encoding genes, as well as the phylogenetic relationships between strains. Phylogenetically, strain relationships are similar using average nucleotide identity (ANI) values and multi-gene approaches, although PacBio sequencing revealed multiple copies of the 16S rDNA gene which lessened utility at the strain level. Based on ANI values, the New Zealand isolates were distant from other isolates and may represent a new species. While all of the genomes examined shared some putative toxicity or virulence related proteins, many specific genes were only present in a subset of strains.

## Highlights

• We examined genomes of 11 *Brevibacillus laterosporus,* a bacterium which is antagonistic to invertebrates and/or microbes
• Multiple phylogenetic methods showed New Zealand isolates more distant than all other isolates
• Each genome could contain 11–13 copies of the 16S rDNA gene, some of which were not identical
• Many putative toxin encoding genes were present in the genomes, but the toxin complement varied from isolate to isolate
• Variation in occurrence of toxin-encoding genes indicates the potential to find strains with new combinations of activities

## 1 Introduction

*Brevibacillus laterosporus* is a gram positive, spore forming, globally ubiquitous bacterium of soil and water [34]. Originally described as *Bacillus laterosporus* [47], the genus was distinguished from the genus *Bacillus* by Shida et al. [72], based on 16S rDNA sequences. Strains of the bacterium have been reported as pathogens of a range of invertebrates, as well as antagonists of other microorganisms, due to the production of antimicrobial molecules and other secondary metabolites [66]. *Brevibacillus laterosporus* is therefore an important resource for the bio-control of several globally important pests and diseases.

Over recent years, a surprising diversity of toxin activities has been reported across strains. A well-established activity of *B. laterosporus* is insecticidal activity against some Diptera [24,27,66], Lepidoptera [24,84] and Coleoptera [61]. Furthermore, it has been reported by De Oliveira et al. [24] that the fresh water snail *Biomphalaria glabrata* is highly sensitive to a *B. laterosporus* strain*.* Recently, nematocidal activity of the bacterium has been described [37] and confirmed by Zheng et al. [99] who found that all four strains they tested were active against nematodes.

Activity of some strains against microorganisms has also been reported. For example, *B. laterosporus* AMCC100017 is active against *Streptomyces* spp., the causative agent of potato common scab (PCS) [18]. The strain was also noted as a rhizosphere colonizer [18], although no function was correlated with this observation. Strain B4 has been found associated with the rice rhizosphere and has been reported to reduce the occurrence of bacterial brown stripe of rice caused by *Acidovorex avenae* subsp. *avenae* [39]. Antifungal activity has also been shown against some phytopathogens [76,100], and a probiotic effect of some *B. laterosporus* strains has even been suggested [36,52,59].

A range of *B. laterosporus* virulence factors, active against various targets, have been identified. Marche et al. [50] reported that four spore surface located (associated with the spore coat and canoe-shaped parasporal body) proteins of *B. laterosporus* UNISS18 are virulence factors against flies and the nematocidal activity described appears to be related to extracellular protease production by strain G4 [80,81]. Marche et al. [51] further demonstrated that a range of virulence related genes were expressed during pathogenesis of insects, as well as culture, for UNISS18, including chitinases, proteases, bacillolysin, an Mtx toxin and protective antigens. The antimicrobial lipopeptide, brevibacillin, produced by *B. laterosporus* OSY-I1, is antagonistic against gram positive bacteria [90]. The occurrence of these and other virulence factors has not been compared across the known strains of *B. laterosporus*.

Three strains of *B. laterosporus* were recently isolated from plants in New Zealand. Two isolates, 1951 and 1821L, were found in surface sterilized brassica seeds, suggesting an endophytic origin [84]. Another isolate, Rsp, was recovered from a potato plant [10]. All isolates were found to have larvicidal activity against the diamondback moth *Plutella xylostella* ([54,84]. Near complete genomes of these three strains have now been obtained through both short- and long-read sequencing. Two other isolates, NRS590 and CCEB342, have also been sequenced because of their insect toxicity and are also presented in this study for the first time. NRS590 has reported toxicity to *Simulium vittatum* [73], as well as activity against the cigarette beetle *Lasioderma serricorne* (Coleoptera), *Aedes aegypti* and *Culex quinquefasciatus* (Diptera) ([27,61,92]. Isolate CCEB342 has reported activity against *Lasioderma serricorne* [92], Coleoptera [61] and *Culex quinquefasciatus* [27].

Genome sequences are publicly available for a number of other *B. laterosporus* strains: the type strain DSM25 (unpublished GenBank record CP017705.1); LMG 15541 [25]; UNISS 18 (NCIMB 41419) active against Diptera [15]; B9, an antagonist of *Acidovorax avenae* subsp. *avenae* (bacterial brown stripe of rice) from China [48]; PE36, a feral hog associated strain [79]; and GI9, which was recovered from a subsurface soil sample in India and displays antimicrobial properties [70]. Isolate DSM25, listed as the type strain of *B. laterosporus* has two versions of its genome in NCBI, with a full chromosome submitted in October 2017 (unpublished GenBank record CP017705.1, used in our analyses).

There is a high level of interest in commercialisation of strains of *B. laterosporus*, with patents filed on activities as diverse as insect control and microbial fertilisers (e.g. [12,31,33,52,59,65,69]. Given the surprisingly high level of variation in toxin activity across different *B. laterosporus* strains, comparing the genome sequences of a number of strains may help to define the role of genetic regions in pathogenesis. Here, we focus on the distribution of putative toxin and virulence associated genes relative to 16S rDNA and multi-gene phylogenetic relationships within the species, as well as whole genome comparisons. Our aim is to explore the distribution of virulence loci relative to the phylogeny of the species, as constructed from core genes, and identify putatively horizontally acquired regions.

## 2 Methods

### 2.1 DNA preparation and sequencing

Genome sequences of five isolates of *B. laterosporus* are presented here for the first time. Three are from New Zealand: 1951, 1821L [84] and Rsp [10], all with activity against diamondback moth and mosquito larvae. Two other isolates, NRS590 and CCEB342 [27], kindly supplied by the late Allan Yousten, Virginia Polytechnic Institute and State University, USA, were also sequenced.

For the New Zealand strains, DNA was extracted from overnight cultures of *B. laterosporus* grown in LB broth, using the DNeasy Blood and Tissue kit (Qiagen) as per the manufacturer's instructions. For strains CCEB342 and NRS590, DNA was extracted using the QIAmp DNA mini kit (Qiagen) as per the manufacturer's instructions*.*

Three isolates (1951, 1821L and Rsp) were sequenced using PacBio long reads by Macrogen (South Korea), as well as Illumina short-read sequencing at Massey University, New Zealand. Assembly of PacBio sequences was achieved using CANU 1.6 [42] using default settings.

For 1951, 1821L and Rsp, Illumina short reads were assembled with the ABySS 1.3.0 de Bruijn graph assembler. For NRS590 and CCEB342, Illumina short reads were assembled using Velvet 1.2.10 [93] within Geneious 9.0 (http://www.geneious.com, [40]).

### 2.2 Annotation and comparative genomics

Assembled genomes were annotated using the RAST server [2,13], RNAmmer 1.2 [44] and tRNAscan-SE 2.0 [49]. Gene prediction and annotation of genomes submitted to GenBank were performed with the NCBI Prokaryotic Genome Annotation Pipeline (2018 release). Additional BLAST searches were also performed using a custom database constructed with known insecticidal toxins from entomopathogenic bacteria. Whole genome alignments were conducted using Mauve 2.4 [20,21]. The ANI values [32] of the genomes, compared to 1821L as a reference, were calculated using the enveomics ANI calculator (http://enve-omics.ce.gatech.edu/ani/index; accessed December 2017) [63]. Two ANI values were calculated for each genomic comparison, with one genome as the reference and one as the query, and then the converse. The distance matrix from ANI comparisons was used to generate trees using the Neighbor-Joining method [68] in the program T-REX 1 [11].

Core genomes were determined using the Gview server (25 April 2016 version) [58]. A 16S rDNA analysis was carried out using 16S rDNA genes predicted by RAST and RNAmmer [44]. For calculating the maximum likelihood trees of multigene phylogenies, genes were identified using tBLASTn. All gene sequences used are listed in Supplementary Table S1. The identified gene sequences were concatenated and the codons aligned using MUSCLE 3.5. Maximum likelihood trees were produced using MEGA 6 [43]. Phage regions within the genomes were predicted using the PHASTER server (http://phaster.ca/; accessed November 2017) [1,101]. The presence or absence of toxin encoding genes within the genomes was determined using online versions of BLASTx and tBLASTn (https://www.ncbi.nlm.nih.gov/).

### 2.3 Prediction of biosynthetic gene clusters

The analysis of gene clusters potentially involved in the synthesis of secondary metabolites from bacteria was performed with antiSMASH 3.0 [88]. Each draft genome sequence (fasta format) was manually uploaded to the antiSMASH web server (http://antismash.secondarymetabolites.org). Complete genome sequences were directly loaded from GenBank using accession numbers NZ_CP007806, CP011074 and CP017705 for strains LMG, B9 and DM25, respectively.

### 2.4 Alignments

Alignments of protein or DNA sequences were performed in Geneious 9.0 using MUSCLE, unless otherwise stated. Trees were generated using FastTree 2.1.5 using the Jukes-Cantor model. Putative crystal toxin (Cry) proteins were aligned using MUSCLE in the Geneious environment with the UPGMA clustering method and CLUSTALW sequence weighting, using default settings (the first and second iterations used the kmer6–6 distance measure, and the third iteration used the pctid-Kimura distance measure).

The 16S rDNA alignments and multigene analyses were completed using MEGA 6 with the Maximum Likelihood method based on the Tamura-Nei model. Initial tree(s) for the heuristic search were obtained automatically by applying Neighbor-Joining and BioNJ algorithms to a matrix of pairwise distances estimated with the Maximum Composite Likelihood (MCL) approach, and then selecting the topology with the superior log likelihood value. Evolutionary analyses were conducted in MEGA 6 [77].

## 3 Results and discussion

### 3.1 Assembly

Completed genomes used for comparisons and statistics are shown in Table 1. For one newly presented genome 1951, the assembly gave three contigs (chromosome) plus one plasmid. For 1821L, we identified one chromosome plus two plasmids (based on PacBio long-read sequences). However, for Rsp, PacBio sequencing was used to identify the plasmids, but the 123 contigs assembled from Illumina short-read assemblies (ranging from 200 bp to 258,731 bp) were used to identify gene regions. For CCEB342, the genome assembled into 113 contigs ranging from 130 to 483,838 bp, and for NRS590 into 162 contigs ranging from 126 to 267,511 bp, both from Illumina short-read assemblies.

**Table 1:** Features of the *Brevibacillus laterosporus* genomes.

	Strain										
	DSM25	LMG15441	GI9	B9	PE36	UNISS18	1951	1821L	Rsp	CCEB342	NRS590
GenBank Accession No.	CP017705.1	CP007806.1	CAGD01000001-61	JNFS01000001-3	AXBT01000001- 63	MBFH01000001–64	RHPK00000000^b^	CP033461–4^b^.	RHPL00000000^b^	RKQD00000000^b^	RKQC00000000^b^
Genome size (Mb)	5.4	5.11	5.18	3.16^a^	5.13	5.55	5.48	5.56	5.39	4.53	5.29
Contigs	69	1	61	1	62	64	3	1	112	87	112
CDSs	4687	4291	4372	4304	4356	4451	5204	5326	5268	4305	4859
GC%	40.7	41.1	40.8	41.3	41.1	41.1	40.5	40.7	40.9	41.1	40.2
Plasmid No.	–	2	–	2	–	–	1	3	2	1	–
rRNAs	33	36	7	39	3	20	36	36	32	7	7
tRNAs	110	113	100	112	98	58	111	112	94	92	92
Reference	Lee et al. unpublish.	Djukic et al. [25]	Sharma et al. [70]	Li et al. [48]	Theodore et al. [79]	Camiolo et al. [15]	This study	This study	This study	This study	This study

a The B9 deposited sequence includes two large “plasmids” (1.37 Mb and 0.733 Mb), which are both larger than plasmids identified in other *B. laterosporus* strains. Including sequences reported as the large plasmids with the genome results for B9 leads to an overall genome size similar to the other strains, suggesting that these DNA sequences may be genomic regions misidentified as plasmids.

b Bioproject PRJNA503267.

### 3.2 General features of the genomes

The genome features of all available *B. laterosporus* genomes, including the New Zealand isolates, are summarized in Table 1. The genome sizes and the GC content of the New Zealand isolates are similar to those of the other *B. laterosporus* strains. The number of predicted coding sequences (CDS) is, however, greater for the New Zealand strains. Of the newly sequenced non-New Zealand strains, NRS590 had a genome size of 5.30 Mb, similar to the other strains, while the CCEB342 assembled genome was found to be smaller than the other *B. laterosporus* genomes sequenced here at 4.54 Mb. One other sequenced genome, B9, was only 3.16 Mb but had two large “plasmids” of 1.40 and 0.733 Mb (Table 1), which, taken together with the 3.16 Mb genome, would equate to a similar size as the other genomes (5.29 Mb).

### 3.3 Whole genome alignments and average nucleotide identity (ANI)

ANI is a measure of the pairwise genomic similarity between coding regions of two genomes at the nucleotide level. An ANI > 95% is often taken to mean that the genome pair are members of the same species [32,41]. The use of ANI values has been proposed as the most workable alternative to the use of DNA-DNA hybridization [82], and an ANI value of approximately 94% has been suggested to represent the accepted 70% DNA-DNA hybridization score used for species delineation [41].

**Fig. 1:**
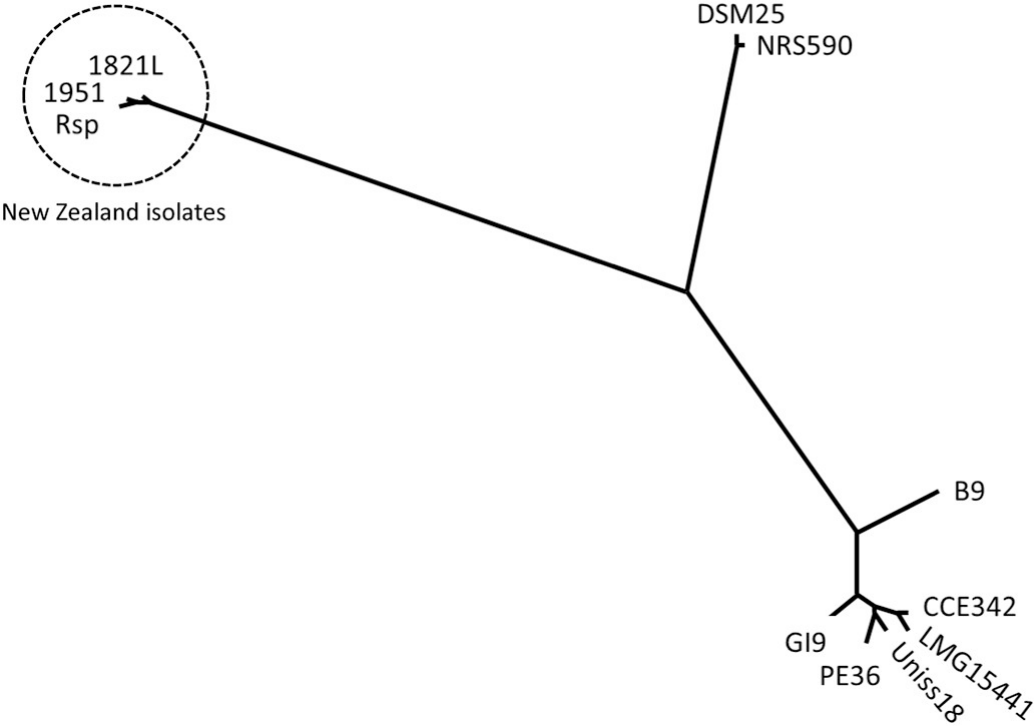
Proportional phylogenetic tree from average nucleotide identity comparisons (Neighbor-Joining method). Pairwise comparison distance matrix of ANI values in Supplementary Table S2.

ANI calculations indicate that the New Zealand strains of *B. laterosporus* are distinct from the other strains. The similarity between all New Zealand strains was very high at approximately 99% (Supplementary Table S2 and Fig. 1). The RAST and PHASTER analyses identified that the regions differing between the three genomes are mainly located within three phage-related regions.

Visual representation of the ANI distance matrix (Fig. 1) shows the clusters of strains. The New Zealand strains (1821L, 1951, Rsp) are distant to other strains with <88% identity. DSM25 and NRS590 were very similar (>99%) to each other, but <90% identical to all other strains. The remaining strains clustered together, although B9 was more distant to all other strains in this cluster, even with the inclusion of the putative mega-plasmids. Alignments of groups of genomes using MAUVE (Fig. S1) illustrate the conservation of gene regions.

### 3.4 Phylogeny

#### 3.4.1 16S rDNA

The most common phylogenetic comparisons of bacteria have typically used the single 16S rDNA gene [91]. However, the presence of multiple 16S rDNA genes in many bacteria and the difficulty of using a single gene to determine phylogenetic relationships have resulted in more recent studies taking a multiple-gene approach [46]. For example, Berg et al. [6] recently showed that 16 s rDNA sequences are insufficient to distinguish between *B. laterosporus* and *Paenibacillus larvae,* both common in beehives.

**Fig. 2:**
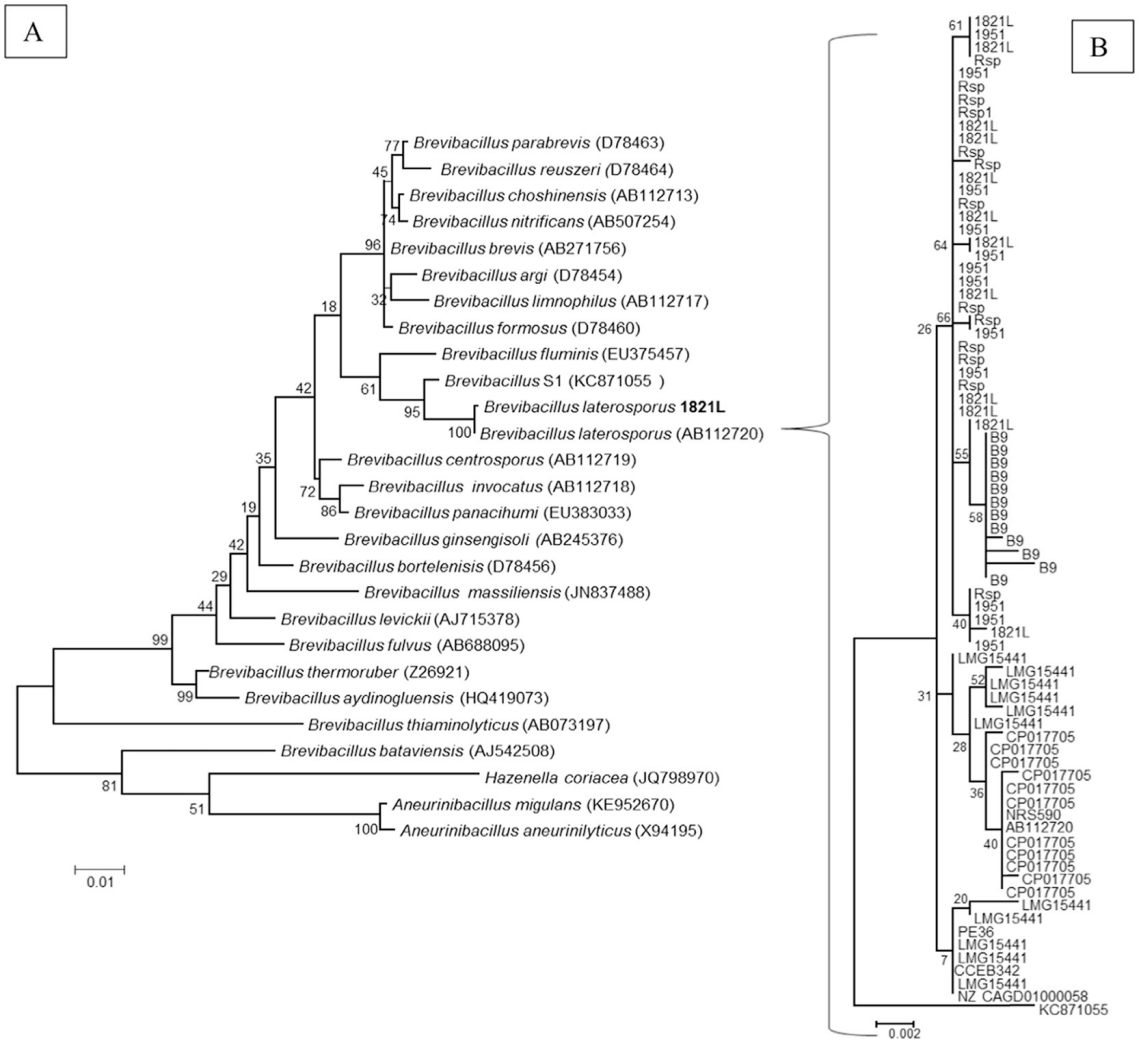
Trees generated from alignment of 16S rDNA sequences of *Brevibacillus laterosporus* genomes. A) Placement of 1821L (New Zealand) in the tree from Zheng et al. [98]. B) Alignment of all 16S rDNA gene sequences. Bar = A) 0.01 nt substitutions per site and B) 0.002 nt substitutions per site.

Previously, Vetrovsky and Baldrian [85] studied within-genome variation in the copy number and sequence of the 16S rDNA gene across a range of bacteria. They found limited variation and low copy number in most groups. However, some species, including *Bacillus* spp., showed high intraspecific variability in 16S rDNA copy numbers, with up to 14 copies in some genomes. We found 11–13 copies of this gene in the fully sequenced genomes of *B. laterosporus* (Rsp 12–13 copies [two regions were on the ends of PacBio unitigs]; 1821L 1951, B9 12 copies; LMG15441, DSM 25, 11 copies). Conversely, some strains (GI-9, PE36 and UNISS18) were represented by only one, incompletely assembled gene in their GenBank records and so are not represented in Fig. 2.

The genomes sequenced using PacBio technology (1951, 1821L and Rsp) allowed the most accurate assessment of the 16S rDNA copy number and clearly demonstrated that the gene repeats are not always identical (see Fig. 2). The 16S rDNA genes were often partially represented at the end of the contigs, reflecting the difficulty of assembling repetitive regions using short-read approaches. In some cases, gene copies are shuffled (e.g., copies of the gene in Rsp span large parts of the tree), which provides further evidence that 16S is not a good phylogenetic marker for this species.

#### 3.4.2 Multi-gene phylogeny

**Fig. 3:**
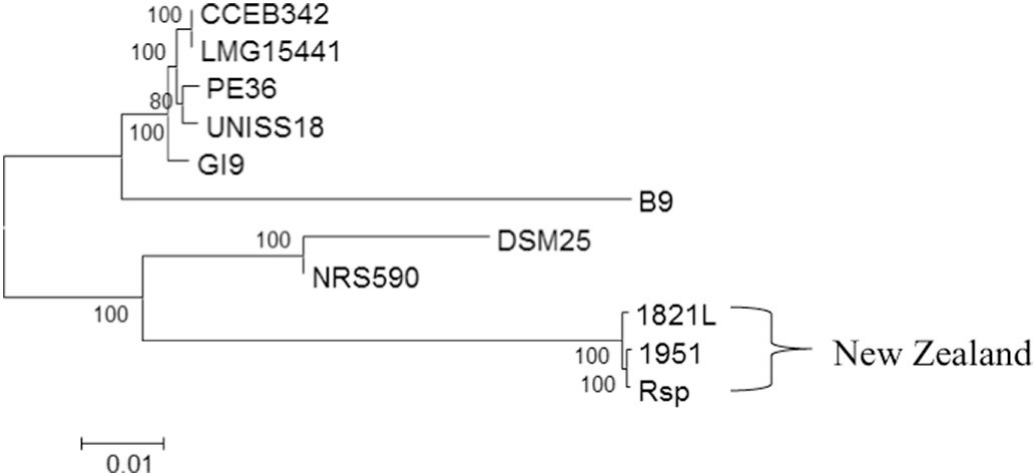
Maximum likelihood multi-gene alignment of concatenated gene sequences prepared using MEGA 6 for 41 genes. Genes used in the analysis are listed in supplementary Table S1 and alignments of each gene group are given in supplementary Fig. S2.

As 16S rDNA analysis is often unable to distinguish between strains within a species, and sometimes not even between species, use of multi-gene approaches has been increasing. Lang et al. [46] found that Maximum Likelihood alignments of concatenated sequences of 24 genes was an appropriate method for inferring bacterial phylogenies. Several papers have used sets of conserved genes to distinguish between strains and species within the genus *Bacillus*. Rocha et al. [62] used eight reference genes, Lan et al. [45] used 10 marker genes specifically chosen for *Bacillus*, and Lang et al. [46] used 24 genes including some included in the other sets. Using each set of conserved genes (Supplementary Table S1) showed similar relationships between the *B. laterosporus* genomes (Supplementary Fig. S2), which was also similar to the relationships observed when all genes were concatenated (Fig. 3). The phylogenetic trees generated were similar in most respects to the ANI analysis (Fig. 1) with regard to relationships between strains, especially for the New Zealand strains. The three New Zealand strains, 1821L, 1951 and Rsp, were consistently grouped together but were mostly distinct from the other strains. CCEB342 and LMG15441 grouped together, usually close to the PE36 and UNISS18 pair, with GI9 in the same cluster. DSM25 and NRS590 were on the same sub-branch, but more distinct from each other. Although B9 generally fell on the branch with the major non-New Zealand strain group, it was consistently more distant.

### 3.5 Toxin gene distribution

Strains of *B. laterosporus* have demonstrated insecticidal and nematicidal activity against a range of insects and invertebrates, as well as producing a broad range of antimicrobial molecules [66]. Insecticidal activity has been attributed to a range of toxins. For example, the production of parasporal crystals has been associated with activity against mosquitoes [53] and several crystal toxin proteins from *B. laterosporus* have been patented for this application [12]. The effect of *B. laterosporus* on treated houseflies has also been well described [67]. The bacterium targets the midgut of infected flies causing changes to the cells such as the alteration of microvilli, cytoplasmic vacuolization and changes to the mitochondria. Such toxin-cell interactions are also important in the mode of action of *Bacillus thuringiensis* δ-exotoxins against other insects [67]. The expression of an Mtx toxin, homologous to Cry75Aa, in the housefly active strain UNISS18 has been shown [51]; Some strains of *B. laterosporus* are active against nematodes via the degradation of their cuticle by proteases such as BLG4 [80]. Nematocidal activity in one strain was correlated with a range of proteins common to nematocidal bacilli [99].

We used putative and known toxin proteins, which included representatives of each Cry protein at the primary rank, as the reference in BLASTp comparisons between the genomes. These include Cry1-Cry75 (Supplementary Table S3), Cyt proteins at primary rank (Cyt1-Cyt3); taken from the Bt nomenclature database (http://www.lifesci.susx.ac.uk/home/Neil_Crickmore/Bt/), and a range of other toxins and virulence factors (Supplementary Table S4). Table 2 lists the distribution of homologs of the reported putative toxin or virulence related proteins predicted in the *B. laterosporus* genomes studied in this work. We have highlighted putative toxins through homology, rather than through experimentally determined function. All genomes encoded homologs of thiol-activated cytolysin, Etx/Mtx2 family proteins and chitinase (Table 2). Thiol-activated cytolysin is important for the degradation of cholesterol containing membranes, as found in insects. Chitinases break down chitin-containing exoskeleton components of insects and chitin present in fungal cell walls and was shown to be expressed in the insect haemolymph by *B. laterosporus* UNISS18 [51].

**Table 2:** Summary of the putative toxin protein distribution predictions within the *Brevibacillus laterosporus* genomes. Values are pairwise identity to the representative predicted whole protein sequence in column 3 (GenBank accession). Where two values are shown, two genes were present.

		Strain Pairwise Identity (%)											
Search protein used	Function	GenBank Accession No.and organism source.	LMG-15441	1951	1821L	Rsp	PE36	B9	GI9	UNISS18	CCB342	NRS590	DSM25
ETX/MTX2	Etx/Mtx2 family beta pore forming toxin	WP_022584503.1 *B. laterosporus* PE36	99	97	97/97	97	100	98	99	100	99	98	93/98
ETX MTX2	Etx/Mtx2 family beta pore forming toxin	WP_022584953.1 *B. laterosporus* PE36	97	–	26	–	100	92	98	98	97	87^a^	87
MTX4	Etx/Mtx2 family beta pore forming mosquitocidal toxin	WP_080717387.1 *Lysinibacillus sphaericus*	–	36	33	36/36/36	–	–	–	–	–	36	37
Plx2B	Protective antigen-like protein	AGJ74030.1*Paenibacillus larvae*	–	61/62	70/61/61/24	61/63	–	–	–	–	–	–	–
Isp1a	Insecticidal secreted protein	CAI40767.1*B. laterosporus*	–	–	–	–	–	–	–	–	–	98	97
Isp2a	Insecticidal secreted protein	CAI40768.1*B. laterosporus*	–	–	–	–	–	–	–	–	–	98	98
Isp2b	Insecticidal secreted protein	WP_001996221.1 *Bacillus cereus*	–	55/31	62/62/30	55/31	–	–	–	–	–	–	–
Vip1–4	see Table 3												
LF	lethal factor domain protein: endopeptidase	WP_099327290.1 *B. laterosporus* DSM25	87/45	94	80/95	94	88/45	88/46	89/81/46	89/45	87/45/45	100/90	100/55
ChiA	Chitinase	AKN21157.1*B. laterosporus* M64	99	89	89	89	99	98	99	99	99	93	93
ChiC	Chitinase	AKN79540.1*B. laterosporus* M9	99	88	88	87	99	97	98	99	99	91	90
Alv	Thio-lactivated cytolysin	WP_003335622 *B. laterosporus* LMG 15441	99	96	96	96	99	97	99	99	100	97	96
Pebl1 A60	Protein elicitor	AJE60449.1*B. laterosporus* A60	100	–	–	–	100	95	97	100	100	88	100
ExsC	cell wall proteins	AQX44452.1*B. laterosporus* UNISS18	100	53	54	54	100	93	99	100	100	90	90
CHRD	cell wall proteins	AQX44453.1*B. laterosporus* UNISS18	99	–	–	–	100	–	99	100	99	–	–
CpbA	cell wall proteins	AQX44450.1*B. laterosporus* UNISS18	100	86	86	86	100	94	100	100	100	92	92
CpbB	cell wall proteins	AQX44451.1*B. laterosporus* UNISS18	–	–	–	–	99	93	99	99	–	–	–
Cry18Aa	3-domain family Pesticidal crystal protein	CCF16695.1*B. laterosporus* GI9	–	–	75/92	–	–	–	76/99	–	–	–	–
Cry27Aa	3-domain family Pesticidal crystal protein	WP_016098322.1 *Bacillus cereus*	–	45	45	46	–	–	–	–	–	–	–
Cry35Aa1	Toxin 10 family Insecticidal crystal protein	AAG50342.1*B. thuringiensis* PS80JJ1	–	26	–	26	–	–	–	–	–	–	–
BrvA	Brevibacillin synthetase A	ASV51722.1*B. laterosporus* OSY-I1	93	90	90	90	93	93	93	93	93	100	100
BrvB	Brevibacillin synthetase B	ASV51721.1*B. laterosporus* OSY-I1	90	90	90	89	90	91	90	90	90	98	98
BrvC	Brevibacillin synthetase C	ASV51723.1*B. laterosporus* OSY-I1	93	97	97	97	93	93	93	93	93	97	98
BrvD	Brevibacillin synthetase D	ASV51724.1*B. laterosporus* OSY-I1	57	97	98	97	70	70	69	70	86	98	98
BrvE	Brevibacillin synthetase E	ASV51725.1*B. laterosporus* OSY-I1	91	98	98	98	91	91	91	91	91	98	98
BrvF	Brevibacillin ABC transporter	ASV51726.1*B. laterosporus* OSY-I1	97	96	96	97	97	98	97	97	97	100	100
PurL	Amidophosphoribosyl-transferase	NP_388531.2*Bacillus subtilis* str. 168	65	65	65	64	65	65	65	65	65	65	65
EcaA1	calcium-transporting ATPase	XP_572412.1*Cryptococcus neoformans* var. *neoformans* JEC21	36	36	37	35	36	37	36	36	36	37	37
Blg4	alkaline serine protease	AAU81559.2*B. laterosporus* G4	37	36	36	36	37	37	37	37	37	37	37
Enp	extracellular neutral proteases	ABI93802.1*B. laterosporus* G4	39	38	38	34	39	39	39	39	39	39	33

— not present.

a Incomplete sequence.

Some toxins were absent from the genomes of the New Zealand isolates, but were present within the others and vice versa (Table 2). Potential toxin types are detailed below.

### 3.6 Potential insecticidal proteins

**Fig. 4:**
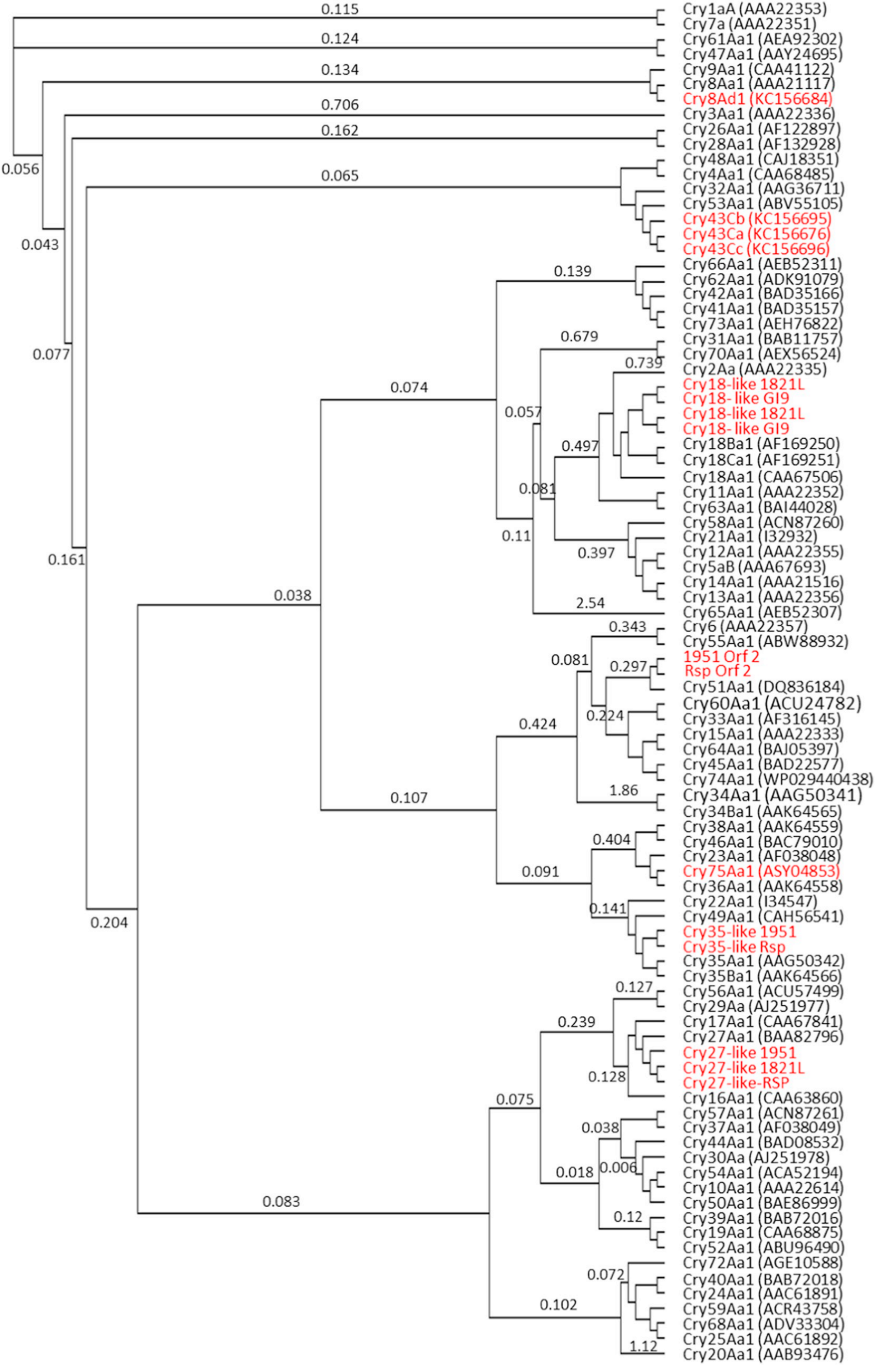
Cry protein phylogeny. In red are Cry proteins from *Brevibacillus laterosporus,* with New Zealand isolates 1821L, 1951 and Rsp labelled*.* Cry 75 and Cry18 were present in GenBank records; Cry43 and Cry8 from *B. laterosporus* were previously reported in a patent [69]. Numbers on branches are substitutions per site. Figure was generated using FastTree in Geneious following MUSCLE alignment using the CLUSTAL algorithm. (For interpretation of the references to colour in this figure legend, the reader is referred to the web version of this article.)

A large array of Cry proteins has been identified across a number of bacteria, including *B. laterosporus,* and are divided into over 78 types (http://www.btnomenclature.info/; accessed August 2018), with more likely to be described. Despite the common Cry name, these proteins belong to a range of distinct structural classes [8] with different modes of action. Using a selection of proteins from each described Cry toxin class (Supplementary Table S3) for comparison, the Cry-like predicted proteins from *B. laterosporus* were compared (Fig. 4).

### 3.7 Three domain proteins

Two Cry proteins, Cry8 and Cry43, representing members of the three-domain toxin family and attributed to strains of *B. laterosporus,* are present in GenBank and listed in the *B. thuringiensis* nomenclature database [19] (http://www.btnomenclature.info/). Neither of these toxin families was identified in the strains studied in this work, but homologs of other three-domain Cry proteins were found in some genomes.

Cry27 has previously been reported to be active against dipteran insects [83]. All three New Zealand isolates have a homolog with approximately 42% identity to Cry27Aa (Fig. 4) but this was absent from other strains in our study. The *cry27*-like genes are located near a *boNT* type cluster (Fig. 4) (see below).

Genes encoding Cry18-like proteins, together with associated open reading frames (ORF) [94,95], were found in *B. laterosporus* genomes of New Zealand strain 1821L and GI9 from India (Fig. 4). Each of these had two Cry18 gene repeats adjacent within the region, which in 1821L resides on a large plasmid. Toxin genes in *B. thuringiensis* are found almost entirely on (usually large) plasmids but in other insecticidal bacteria such as *Lysinibacillus sphaericus*, the majority of toxins are encoded on the genome (with some duplication of *bin* toxin genes on a megaplasmid; [7]). Previously, Cry18A [94,95], B and C ([57]; direct Genbank submissions AF169250_2 and AAF89668.1) and associated ORFs have been described from *Paenibacillus popilliae*, a bacterium that causes a chronic disease in scarabs. The level of identity between the two *B. laterosporus* 1821L Cry18-like proteins and the *P. popilliae* Cry18s range from 54 to 75%, while identity to the GI9 Cry18Aa was 66–92%. Within the 1821L, the two Cry18-like predicted proteins were 75% identical (Supplementary Table S5).

### 3.8 Etx/Mtx2 proteins

The *B. thuringiensis* nomenclature database lists three variants of the Etx/Mtx2 family protein Cry75 protein, all from *B. laterosporus* strains (listed in the submissions as toxic to the western corn rootworm *Diabrotica virgifera virgifera*) and Marche et al. [51] reported a Cry75-like Mtx from UNISS18. Cry75 homologs were absent from genomes examined in this work, although other members of the broader Etx/Mtx2 family were identified in all genomes analyzed. An Etx/Mtx2 protein (WP_022584503.1) previously identified in *B. laterosporus* strain PE36 was found as homologs in all genomes (with two copies present in strain 1821L). A further family member from PE36 identified relatively close homologs in all strains except those from New Zealand, among which only strain 1821L showed a (weakly matching) homolog (Table 2). A further Etx/Mtx2 protein, Mtx4 from L. *sphaericus*, has homologs with low identity in the three New Zealand strains and two other strains (NRS590 and DSM25).

### 3.9 Toxin_10 family proteins

A separate family of beta sheet rich toxins is represented by the Toxin_10 family of proteins [8]. Homologs of the Toxin_10 protein Cry35 were found in two of the New Zealand strains (1951 and RSP) on a 3 kb genomic region identical between the two strains. The Cry35 homologs show lower identity (26%) than other Cry toxin homologs described in the *B. laterosporus* strains studied here. Toxin_10 proteins frequently act as parts of two component toxins such as the L. *sphaericus* BinA/BinB toxin [22], Cry34/Cry35 [26], and Cry48/Cry49 [38], although Cry36 [65] and Cry78 [87] are able to act alone. In the case of the Cry35 homolog genes in the two New Zealand *B. laterosporus* strains, an upstream gene in each encodes a putative protein of ~20 kDa that matches lectin domains in Blastp searches and using HHPRED [75] (Fig. 4, labelled Orf 2). Consistent with this, modelling of this putative protein using I-TASSER [64] suggests that it may fold into a lectin-like domain but with little else in the structure. No obvious ribosome binding site is seen upstream of this gene, which may, therefore, represent a pseudogene. Alternatively, we cannot rule out an operon in which this upstream protein might act with the Cry35-homolog as a binary mate (similar to the BinA/BinB Toxin_10 pair) for the Cry35 homolog proteins or an independent candidate toxin.

### 3.10 ADP ribosylating toxin homologs

ADP ribosylating toxins from different organisms are known by a variety of names. Within the taxonomic class Bacilli, names include Vip1/Vip2 described originally from *B. thuringiensis* and Isp1/Isp2 proteins from *B. laterosporus*. ADP-ribosyl transferase toxins are usually two component (AB) toxins, although, as in the case of Mtx1 from L. *sphaericus,* the two subunits may be derived from a single precursor protein by proteolysis [78]. It is interesting to note, however, that Vip1-like proteins often appear to occur in *B. thuringiensis* genomes without an identifiable Vip2. Similarly, other proteins with a PA14 domain at the N-terminus, Binary_ToxB motifs in the centre and a ricin domain at the C-terminus, with similarity to the protective antigen component of *B. anthracis* toxin such as Vip4 from *B. thuringiensis*, have no known ADP-ribosyl transferase enzymatic partner and no known insect target [17]. Plx2B from *Paenibacillus larvae* is also homologous to the protective antigen group, although a partner ADP-ribosyl transferase subunit has not been identified. The activity of isolated Vip1, Vip4 and Plx2B proteins encoded in genomes is, therefore, not established.

Supplementary Fig. S3 and Supplementary Table S6 show the relationships between protein sequences related to ADP-ribosyl transferases and protective antigen-like proteins that were used in our searches. Distinct groups can be seen in Fig. S3 for (i) the ADP-ribosyl transferase enzymatic component (Lethal factor, domain protein, Vip2, Isp2) and (ii) the protective antigen-like proteins that might be expected to play a role in specificity and cell entry for the toxins (Vip1, Vip4, Isp1, Plx2B).

**Fig. 5:**
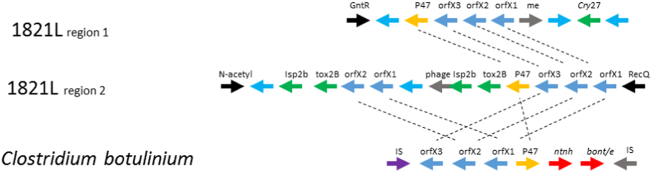
*BoNT* regions from 1821L and a representative region from *Clostridium botulinium* [35]. Blue arrows represent hypothetical proteins; *N*-acetyl-*N*-acetylmuramoyl-L-alanine amidase (EC 3.5.1.28); me – mobile element; Tox2B – Plx2b homology toxin type region with a Binary toxin B core. (For interpretation of the references to colour in this figure legend, the reader is referred to the web version of this article.)

Homologs of the protective antigen family proteins were also found in the genomes. Plx2B homologs with 60–70% identities were found in all the New Zealand strains but no others (Table 2). Isp1a homologs were found in strains NRS590 and DSM25 and homologs of *B. anthracis* protective antigen, Vip1 and Vip4 (e.g., as depicted in Fig. 5), were found in all *B. laterosporus* strains studied here but at lower levels of identity (Table 3). These specificity-determining components of the ADP ribosyl transferases are known to be quite variable.

**Table 3:** Protein similarity of the region shown in Fig. 5 with closest proteins. Each value represents a different predicted protein in the *Brevibacillus laterosporus* genome (Supplementary Table S3).

		Strain Pairwise Identity (%)											
Protein name	Function	GenBank Accession No. and organism source.	LMG-15441	1951	1821L	Rsp	PE36	B9	GI9	UNISS18	CCB342	NRS590	DSM25
	Anthrax toxin protective antigen	WP04119992 *Bacillus anthracis*	40/39	39	39	39	41/40	40/40	40/39	39/39	40/39	40/39	40/39
Vip4Aa1		AQM56941*Bacillus thuringiensis*	30/30	30	30	30	30/30	31/30	30/30	30/30	30/30	30/30	30/30
Vip1	Two component (Vip1/Vip2) Vegetative insecticidal protein, putative receptor binding moiety	AGC08395.1*Bacillus thuringiensis*	26/26	27	27	26	26/27	27/27	25/26	26/27	26/27	27/(27?)	25/26

NRS590: ? indicates possible assemblies of the protein which reside on the ends of three non-identical contigs.

Homologs of the enzymatic ADP-ribosyl transferase family Isp2b protein were only found in the three New Zealand strains (1951, 1821L and RSP; Table 2) in the *boNT* type cluster (see below). Interestingly, Isp2b and Plx2b (toxin 2B) co-occur in the *boNT* cluster twice (Fig. 5) and could be paired. Lethal factor domain protein homologs were present in all *B. laterosporus* strains studied here and Isp1a and Isp2a homologs were found only in strains NRS590 and DSM25. Mtx1 homologs were not found in any strain studied here. These results suggest that all *B. laterosporus* strains described in this report have the necessary apparatus to target cells through an ADP-ribosylation mechanism of action.

### 3.11 *Clostridium boNT* cluster and putative toxin genes

There are two regions in the New Zealand strains of *B. laterosporus* (but in none of the others) that resemble the *Clostridium* botulism gene cluster*-*encoding genes (*boNT*) [35]. The zinc metalloprotease botulinum toxins are associated with a variety of coding sequences orfX1, orfX2 and orfX3, together with a p47 gene, some of which have potential lipid binding roles (orfX1, orfX2, p47) [5]. In the New Zealand *B. laterosporus* strains, one cluster is associated with putative insecticidal toxin encoding genes with ADP-ribosyl transferase features described above (Isp2B and Binary-ToxB); the other cluster is close to the *cry27*-like toxin gene (Fig. 5). OrfX1, 2 and 3 align against the *Paenibacillus dendritiformis* C454 genome sequence [74] and are present in both clusters in the New Zealand *B. laterosporus* strains. The first gene cluster containing many putative ADP-ribosyl transferase insecticidal toxin homologs in both 1821 and 1951 are almost identical (Supplementary Table S7). The cluster shares some genes with the neurotoxin encoding gene cluster present in *Clostridium*. This “*boNT*” cluster has been shown to be horizontally transferred among *Clostridium* spp., but this is the first report of the cluster in an insecticidal bacterium. Clustering of multiple putative insecticidal toxins together with genes that are auxiliary in the *Clostridium* system may suggest a strongly insecticidal gene cassette. There is variation in the gene arrangement between the two *B. laterosporus* loci, but both regions are likely to be important to toxicity. Similarity between the clusters in the three New Zealand strains for the *boNT* clusters were >98.5% for cluster 1 and >96.8% for cluster 2.

### 3.12 Spore surface proteins

A recent study has attributed activity against flies from strain UNISS18 to four cell wall proteins: CpbA, CpbB, CHRD and ExsC [50]. These proteins were present in the spore coat and parasporal body complex, and were shown to have toxicity to house flies. No mode of action has been determined for CpbA and CpbB, although CpbA had the highest toxicity of the four proteins. The *exsC* gene encodes structural components of the exosporium of *Bacillus* spp. Homologs of CpbA and ExsC were present in all genomes examined. The spore surface located protein CHRD has no clear predicted function, but these proteins are homologs of chordin, a bone morphogenesis protein inhibitor (Mache et al. 2017). Homologs of CHRD were encoded by B9, GI9, PE36 and CCEC342, while CpbB was present in those same four strains as well as B9. The identity was lowest for the New Zealand strains compared to UNISS18 (Table 2).

### 3.13 Antimicrobial compounds

*Brevibacillus laterosporus* also produces a range of antimicrobial molecules active against bacteria and fungi. The operons for Brevibacillin synthetase were present in all genomes and were over 90% identical; the exception being BrvD, which was more variable across the strains. Brevibacillin is a cationic lipopeptide that is active against other bacteria [90] and can disrupt cytoplasmic membranes of yeast [89].

Strain A60 has been registered as a microbial fertiliser in China [86]. This strain has activity against a range of plant pathogens, and a short 11-amino-acid peptide was shown to have direct anti-microbial activity [96]. This short peptide was not present in any of the genomes analyzed. Also, a novel protein elicitor, Pebl1, was characterized from the A60 strain by Wang et al. [86]. The heterologously expressed protein was able to induce a typical hypersensitivity response (HR) and systemic resistance in the tobacco plant *Nicotiana benthamiana.* Plants treated with the protein demonstrated strong resistance to infection by a tobacco mosaic virus (expressing green fluorescent protein, GFP) and *Pseudomonas syringe* pv. *tabaci* compared to untreated plants [86]. The protein Pebl1 was not encoded within the New Zealand isolate genomes, but was found in all of the other strains (Table 2). The mechanism of Pebl1 has not been described, but it is thought that it may act via interfering with the cell membrane of the target cell, leading to the formation of transmembrane channels. Such membrane disruption may lead to the translocation of peptides that may disrupt protein synthesis or impact DNA and RNA [14].

Using the program antiSMASH, each genome was analyzed for the presence of biosynthetic clusters (Table 4). All *B. laterosporus* strains had a cluster highly similar to that previously described for Petrobactin and Basiliskamides, with the exception of DSM25 for the latter. Petrobactin has been shown to be required for growth in iron-depleted conditions in *B. anthracis* and also for full virulence [16]. Metal acquisition is crucial for pathogenesis in many systems [56].

**Table 4:** Detection of biosynthetic cluster using antiSMASH (% of genes similar to known cluster).

Most similar known gene cluster	Strain										
	LMG	1951	1821	RSP	UNISS18	PE36	B9	GI9	CCEB342	NRS	DM25
Petrobactin	100	100	100	100	100	100	100	100	100	100	100
Basiliskamides	95	86	18/9	68/22	68/22	95	95	100	90	81	18
Paenilarvins	50	75								50	
Paenibacterin	40		60/40	40	60/33	60/33	60/60	60	40/33	33	60/40
Polymyxin			40								
Tridecaptin			40	40				40			
Tyrocidine	25/18/18	31/18	25/18/18/18	31/12	31/18	31/13	18	31	18	25/25	25/18/18/12
Pelgipeptin		25		25			50/25	25			
Bacillomycin		20		20						20	20
Fengycin	13				13		13	13	13		20
S-layerglycan	13/8	14	14	14	14	14	14	14	14	14	13
ZwittermycinA	7	44/11/7	29/7/7/7/7/7	44/11/7	7/7	7/7	44/7	11/7	7/7	44/7	29/7/7
Bongkrekicacid				14							
ThurincinH					30	30					
Welwitindolinone		6					6	6	6	6	
Calyculin					20		20				
Yersiniabactin								2			
Difficidin											20
Nostophycin	27										
Mupirocin						13				13	13
Lichenysin			14								
Misakinolide			12								
Nosperin											15
Total detected clusters	12	12	19	14	13	11	13	12	10	12	17

Basiliskamides A and B originally shown to be produced by a *B. laterosporus* isolate, exhibited antifungal activity against *Candida albicans*, an opportunistic human pathogen [4]. For *B. laterosporus* PE36, Basiliskamides A and B inhibited the growth of *Staphylococcus aureus* [79].

### 3.14 Plasmid diversity

Plasmids often carry virulence determinants in bacteria, including *B. thuringiensis* [97]. In *B. laterosporus*, not all strains have identified plasmids, while others have one, two or three (Table 5). As stated above, the “plasmids” in B9 may have been incorrectly identified.

**Table 5:** Plasmids in *Brevibacillus laterosporus.*

Strain	Plasmid designation	GenBank accession number	Size (bp)	Features
1821L	p1821L01		130,971	Cry18 and CRISPR regions. Partial homology with CCEB342 chromosome?
1821L	p1821L02		60,496	RepA homologous with Rsp plasmid 2
1821L	p1821L03		8531	RepA homologous with pBla07 of LMG15441 (poor sequence quality)
1951	p195101		8721	RepA homologous with pBla07 of LMG15441
B9	unnamed plasmid 1	CP011075	733,520	Chromosome?
B9	unnamed plasmid 2	CP011076	1,376,691	Chromosome?
LMG15441	pBrla33	NZ_AFRV01000009	32,617	
LMG15441	pBrla07	NZ_AFRV01000010	7095	RepA homologous with pBla07 of LMG15441
Rsp	pRsp01		8738	RepA homologous with pBla07 of LMG15441
Rsp	pRsp02		63,331	RepA homologous with 1821L plasmid 2 but few other gene regions
CCB342	pCCEB34201		7095	RepA homologous with pBla07 of LMG15441

One group of plasmids ranging from 7 to 8 kb, shared a homologous RepA gene (Table 5). The large plasmid in 1821L, p1821L01, contains both a truncated CRISPR-Cas type I region and the two copies of the Cry18-like protein encoding region.

There are five strains that have 7–8 kb plasmids, which share RepA homologs. Alignment of these plasmid sequences showed that the plasmids pBlar07 from LGM15441 and pCCEB34201 from CCEB342 were identical, but only around 54% identical in DNA sequence to the New Zealand strain plasmids of similar size (pRsp01, p195101, p1821L03) (Supplementary Table S8). The main difference between these small plasmids in the strains of New Zealand and European origin was a DNA repair exonuclease gene-containing an insert of approximately 900 bp in the New Zealand strain borne plasmids. No obvious toxin genes were found on these small plasmids, but the large p1821L01 plasmid from strain 1821L had two copies of the *cry18* toxin encoding gene.

## 4 Conclusions

In this study, we used the growing number of whole genome sequences of the insecticidal, nematocidal and antimicrobial species *B. laterosporus*, to examine the relationships between strains and corresponding distribution of genes potentially involved in antagonistic activity. Comparison of the strains of *B. laterosporus* based on 16S rDNA, multi-gene phylogenies and whole genome comparisons shows that the New Zealand strains were the most distant from the other eight strains examined. The 16S rDNA comparisons showed that most annotated *B. laterosporus* genomes contain 11–13 copies of the 16S rDNA gene, some of which are not identical. While variation in 16S rDNA gene sequences was not found to be so diverse as to be an inappropriate method of species determination, it does mean that it is not a good target for strain identification.

We used a total of 41 genes present in all isolates to construct a multi-gene phylogeny and compared this to an average nucleotide identity (ANI) matrix based on whole nucleotide content. In general, the two approaches returned similar clusters. The ANI result, in particular, suggested that the New Zealand strains 1821L, 1951 and Rsp were different from other strains at a level that could represent a separate species, or at least a clear subspecies. The genome size of all strains was generally in the range of 4.5–5.6 Mb and the GC content (~41%) was very similar, as was expected.

*Brevibacillus laterosporus* strains are antagonistic towards a range of organisms, from microbes to invertebrates. However, our analysis of the presence of genes encoding homologs of known toxins in each genome indicate that most activities are not conserved across the isolates. While all genomes examined shared some putative toxicity or virulence related proteins, many specific genes were limited to subgroups of strains. This variation within a single species shows the potential of this species as biocontrol or even biofertilizers has not been fully explored. Identification to species level is clearly insufficient to determine likely attributes.

Several of the *B. laterosporus* toxin types examined were present in all strains, including thiol-activated cytolysin, ETX/Mtx2, Vip 4-like, chitinase, CHRD, CpbA and EcaA1. Some genes encoding toxins were not present in the New Zealand isolates but were present in all the others, including subtilisin-like serine protease and PEBL1 A60.

The variation in occurrence of toxin-type genes is not surprising in soil-inhabiting bacteria, but does indicate the potential to find strains with new combinations of activities. Increasingly, companies developing bio-control options, such as formulating bacteria as bio-pesticides, want broad-spectrum activity [30]. This study shows that examining more strains is likely to provide new activity combinations.

## Appendix A Supplementary data

Supplementary material Image 1
