# Supplementary material for: Phylogenetic determinants of toxin gene distribution in genomes of Brevibacillus laterosporus
Source: Genomics. 2020 Jan;112(1):1042–53. doi: 10.1016/j.ygeno.2019.06.020 (PMC6978878; doi:10.1016/j.ygeno.2019.06.020)
Supplement: Supplementary file 1 — Supplementary material [file mmc1.docx]

**Supplementary data**

**Supplementary Table S1: Genes used in multiple gene alignment tree analysis**

| Gene name | Protein name | GenBank accession number |
| --- | --- | --- |
| Lang et al. | | |
| *rplB* | 50S ribosomal protein L2 | WP_003333792.1 |
| *rpsI* | 30S ribosomal protein S9 | WP_031413227.1 |
| *rplE* | 50S ribosomal protein L5 | WP_003333782.1 |
| *rpsG* | 30S ribosomal protein S7 | COZIH4.1 |
| *lepA* | GTP-binding protein LepA | SFK05158.1 |
| *infB* | Translation initiation factor IF-2 | WP_007786836.1 |
| *rpsK* | 30S ribosomal protein S11 | COZIK6.1 |
| *rplP* | 50S ribosomal protein L16 | WP_018673893.1 |
| *rpsH* | 30S ribosomal protein S8 | WP_003333780.1 |
| *rplC* | 50S ribosomal protein L3 | WP_012683983.1 |
| *rplN* | 50S ribosomal protein L14 | WP_049742236.1 |
| *rpsC* | 30S ribosomal protein S3 | WP_003333788.1 |
| *rpsE* | 30S ribosomal protein S5 | WP_055746222.1 |
| *rplF* | 50S ribosomal protein L6 | WP_007716264.1 |
| *rpsS* | 30S ribosomal protein S19 | WP_003333791.1 |
| *rpsB* | 30S ribosomal protein S2 | WP_003341356.1 |
| *rplK* | 50S ribosomal protein L11 | WP_023558754.1 |
| *rplD* | 50S ribosomal protein L4 | WP_007716242.1 |
| *rpsQ* | 30s ribosomal protein S17 | WP_003333785.1 |
| *rpsM* | 30S ribosomal protein S13 | WP_018673882.1 |
| *pheS* | Phenylalanyl-tRNA synthetase α subunit | WP_031410890.1 |
| *rplO* | 50S ribosomal protein L15 | WP_003333776.1 |
| *rpsJ* | 30S ribosomal protein S10 | WP_003333796.1 |
| *rpsL* | 30S ribosomal protein S12 | WP_003333803.1 |
|  |  |  |
| Lan et al. |  |  |
| *leuS* | Leucyl-tRNA ligase | BAA35289.1 |
| *pheT* | Phenylalanyl-tRNA synthetase β subunit | BAA15481.1 |
| *tmk* | Thymidylate kinase | BAA35905.1 |
| *ksgA* | Dimethyladenosine transferase | BAE76038.1 |
| *topA* | DNA topoisomerase 1A | BAA14811.1 |
| *cdsA* | Phosphatidate cytidylyl-transferase | BAA77850.2 |
| *glyA* | Serine hydroxymethyl transferase | BAA16459.1 |
| *hisS* | Histidyl-tRNA ligase | BAA16401.1 |
| *infB* | Translation initiation factor IF-2 | BAE77214.1 |
| *pheS* | Phenylalanyl-tRNA synthetase α subunit | BAA15482.2 |
|  |  |  |
| Rocha et al. |  |  |
| *adk* | Adenylate kinase | WP_048035662.1 |
| *gyrB* | DNA gyrase subunit B | WP_007779820.1 |
| *ftsZ* | Cell division protein | WP_048033743.1 |
| *gmk* | Guanylate kinase | AIG27736.1 |
| *glnA* | Type I glutamate ligase | WP_031413534.1 |
| *recA* | DNA recombinase A | WP_048033393.1 |
| *recF* | DNA replication and repair protein F | BAH40981.1 |
| *secA* | Accessory Sec system translocase A2 | WP_029100279.1 |

**Supplementary Table S2.**  Percentage of pair-wise nucleotide similarity (calculated by ANI)

|  | **1951** | **1821L** | **Rsp** | **LMG15541** | **B9** | **GI9** | **PE36** | **Uniss18** | **CCES342** | **NRS590** | **DM25** |
| --- | --- | --- | --- | --- | --- | --- | --- | --- | --- | --- | --- |
| 1951 |  | 99.33 | 99.17 | 84.76 | 84.96 | 84.47 | 84.62 | 84.66 | 84.78 | 86.89 | 86.96 |
| 1821L |  |  | 98.88 | 84.94 | 85.53 | 84.64 | 84.65 | 84.56 | 85.15 | 87.23 | 87.00 |
| Rsp |  |  |  | 84.68 | 84.90 | 84.42 | 84.54 | 84.56 | 84.67 | 86.70 | 86.83 |
| LMG15441 |  |  |  |  | 96.60 | 98.59 | 98.95 | 98.85 | 99.96 | 89.37 | 88.62 |
| B9 |  |  |  |  |  | 96.57 | 96.64 | 96.57 | 96.66 | 89.18 | 89.27 |
| GI9 |  |  |  |  |  |  | 96.64 | 98.57 | 98.69 | 88.50 | 88.51 |
| PE36 |  |  |  |  |  |  |  | 98.91 | 98.96 | 88.81 | 88.80 |
| Uniss18 |  |  |  |  |  |  |  |  | 98.89 | 88.66 | 88.68 |
| CCES342 |  |  |  |  |  |  |  |  |  | 88.76 | 88.70 |
| NRS590 |  |  |  |  |  |  |  |  |  |  | 99.86 |

A


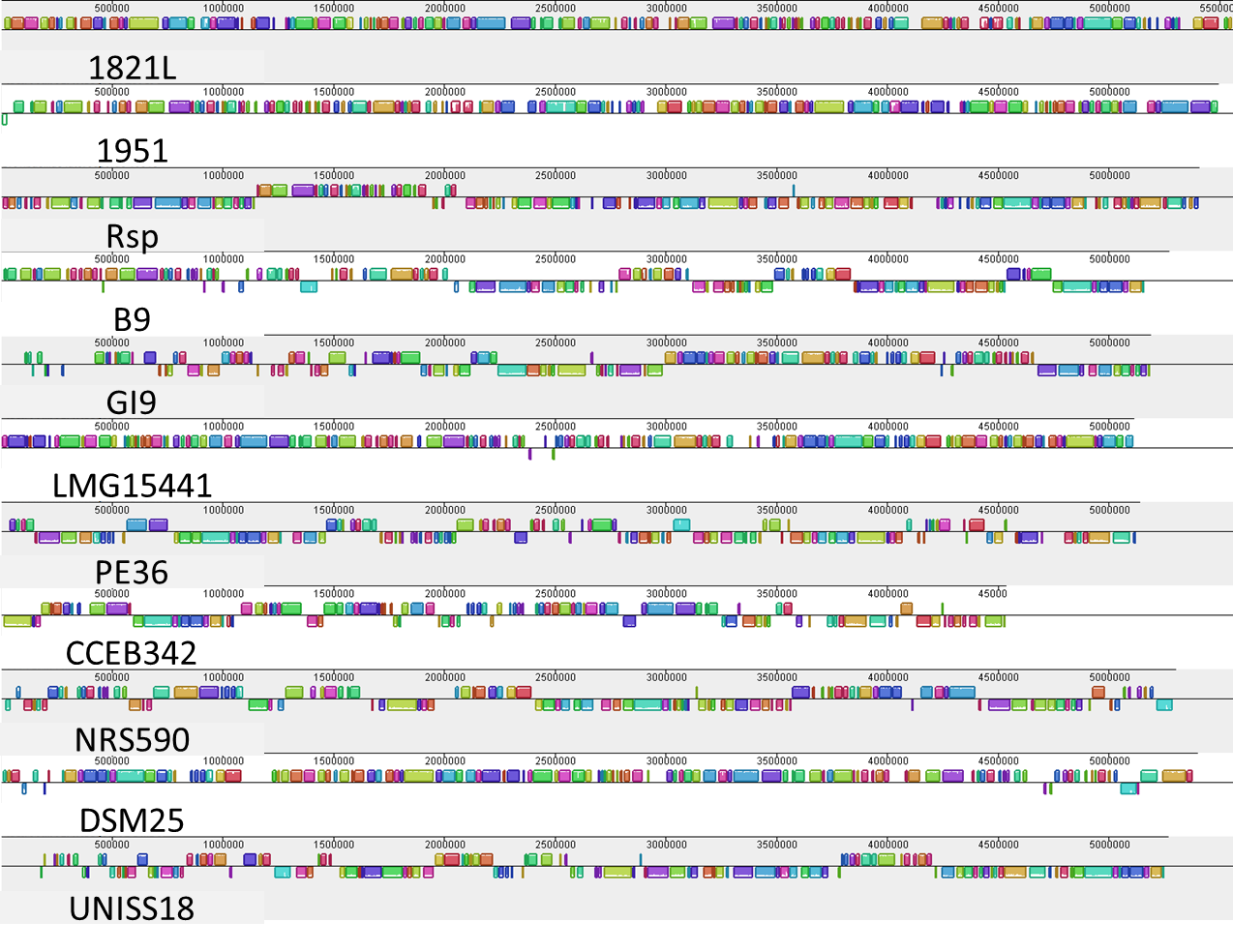


B

**
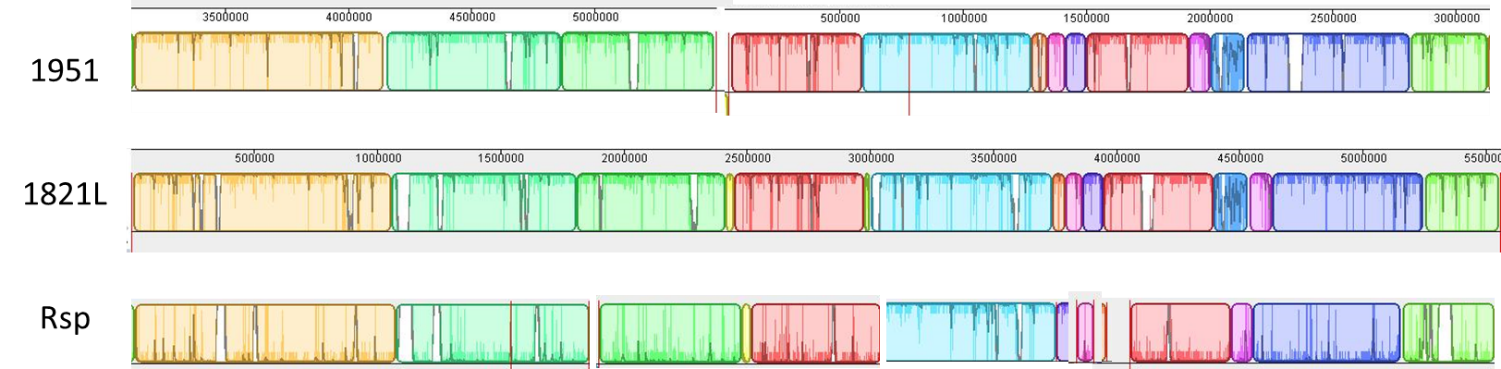
**

C

**
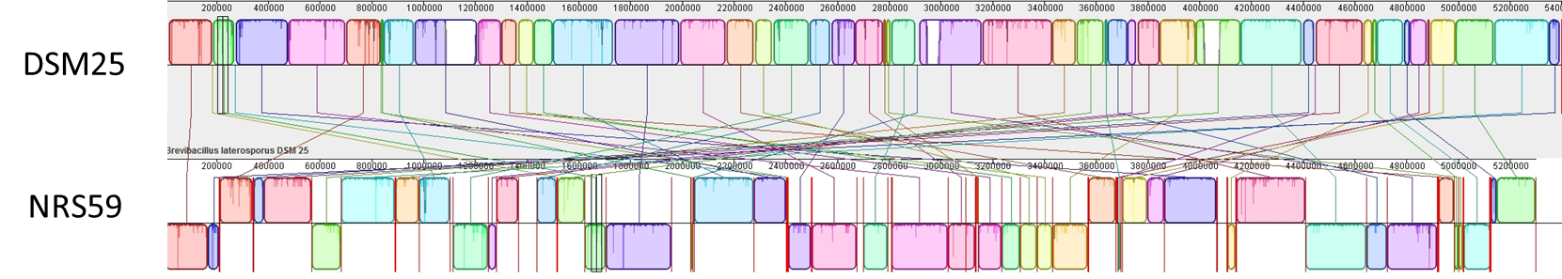
**

D

**
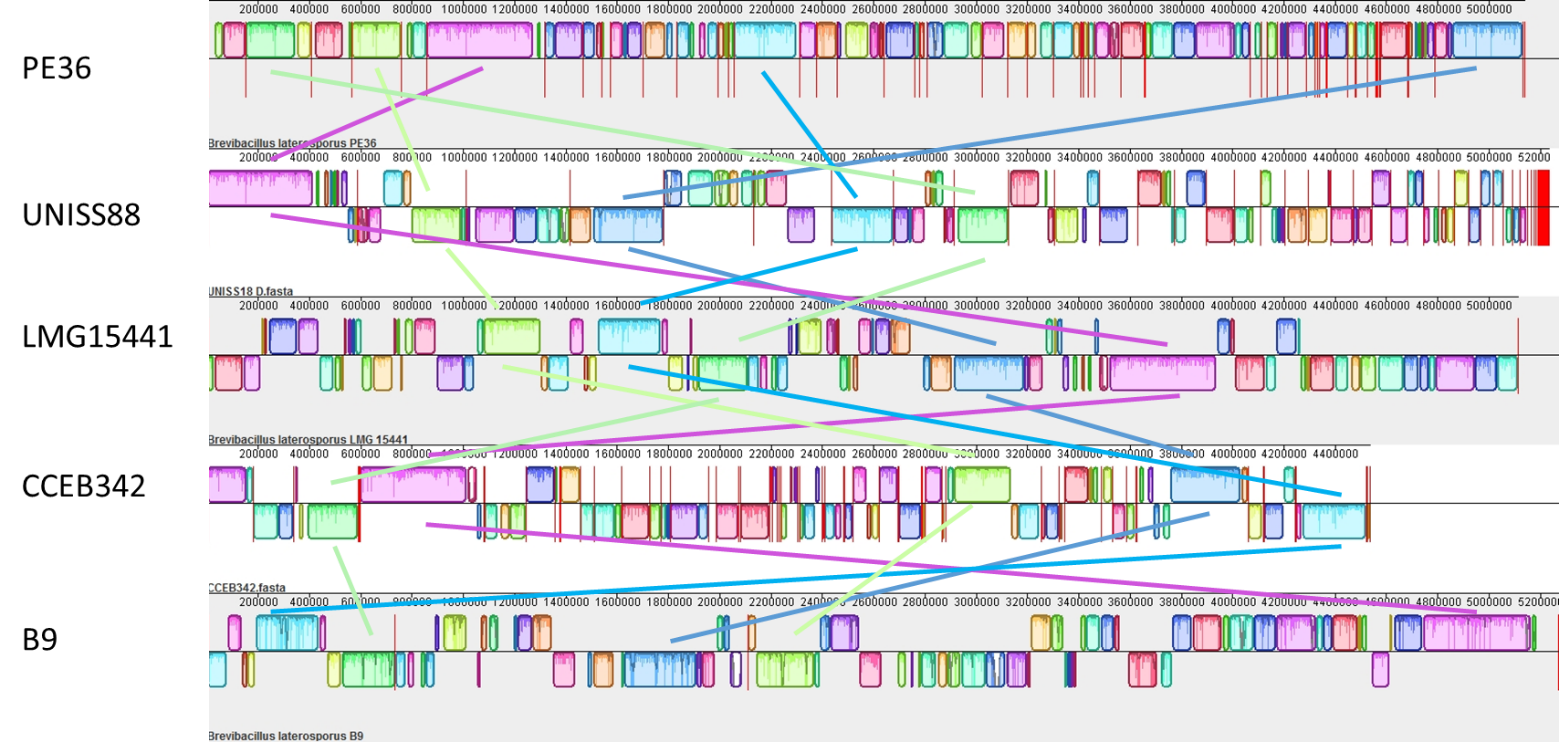
**

**Figure S1:** A) Multiple alignment of conserved genomic sequence with rearrangements (MAUVE) alignment of the *Brevibacillus laterosporus* genomes. B) New Zealand strains composite alignments. C) DSM25 and NRS590 alignment blocks. Colours indicate blocks of homologous DNA. Red lines delimit contigs. D) Grouping of *B. laterosporus* isolates, with largest homologous blocks joined by lines. The direction of similarity is shown by the orientation of the boxes above and below the line. Gene direction is shown by above and below the line boxes.


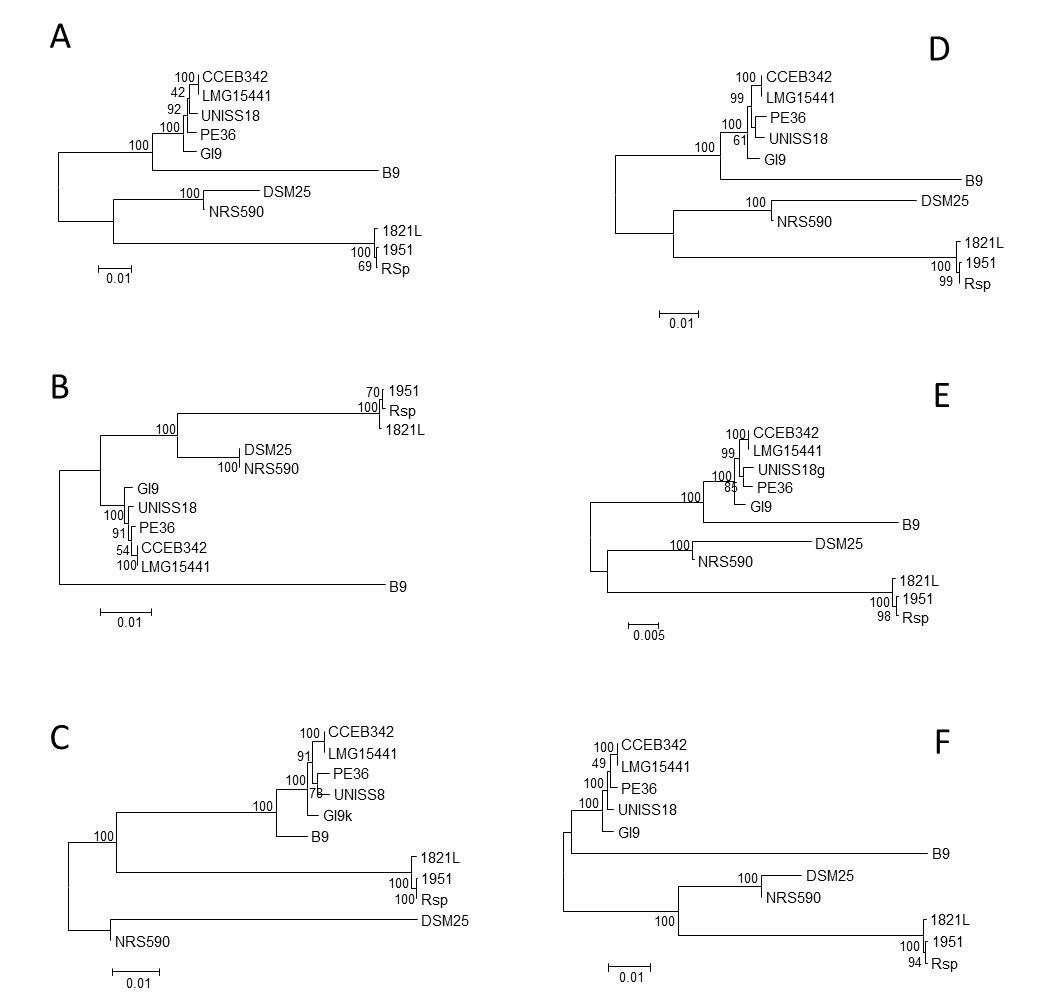


**Supplementary Figure S2**: Maximum likelihood multigene alignment trees of concatenated gene sequences prepared using MEGA 7. **A.** Ten marker genes for *Bacillus* (Lan et al., 2016)*.* **B.** 24 conserved, single copy marker genes (Lang et al., 2013). **C.** Eight bacterial reference genes (Rocha et al., 2015). **D.** The combined genes from A and C, totaling 18 genes. **E.** The combined genes from B and C, totaling 32 genes. **F.**  Combined genes from A and B.

**Supplementary Table S3. Source of comparative Cry protein sequences**

| Protein name (GenBank accession number) | Protein name (GenBank accession number) | Protein name (GenBank accession number) |
| --- | --- | --- |
| Cry1aA (AAA22353) | Cry25Aa1 (AAC61892) | Cry48Aa1 (CAJ18351) |
| Cry2Aa (AAA22335) | Cry26Aa1 (AF122897) | Cry49Aa1 (CAH56541) |
| Cry3Aa1 (AAA22336) | Cry27Aa1 (BAA82796) | Cry50Aa1 (BAE86999) |
| Cry4Aa1 (CAA68485) | Cry28Aa1 (AF132928) | Cry51Aa1 (DQ836184) |
| Cry5aB (AAA67693) | Cry29Aa (AJ251977) | Cry52Aa1 (ABU96490) |
| Cry6 (AAA22357) | Cry30Aa (AJ251978) | Cry53Aa1 (ABV55105) |
| Cry7a (AAA22351) | Cry31Aa1 (BAB11757) | Cry54Aa1 (ACA52194) |
| Cry8Aa1 (AAA21117) | Cry32Aa1 (AAG36711) | Cry55Aa1 (ABW88932) |
| Cry8Ad1 (KC156684) | Cry33Aa1 (AF316145) | Cry56Aa1 (ACU57499) |
| Cry9Aa1 (CAA41122) | Cry34Aa1 (AAG50341) | Cry57Aa1 (ACN87261) |
| Cry10Aa1 (AAA22614) | Cry34Ba1 (AAK64565) | Cry58Aa1 (ACN87260) |
| Cry11Aa1 (AAA22352) | Cry35Aa1 (AAG50342) | Cry59Aa1 (ACR43758) |
| Cry12Aa1 (AAA22355) | Cry35Ba1 (AAK64566) | Cry60Aa1 (ACU24782) |
| Cry 13Aa1 (AAA22356) | Cry36Aa1 (AAK64558) | Cry61Aa1 (AEA92302) |
| Cry14Aa1 (AAA21516) | Cry37Aa1 (AF038049) | Cry62Aa1 (ADK91079) |
| Cry15Aa1 (AAA22333) | Cry38Aa1 (AAK64559) | Cry63Aa1 (BAI44028) |
| Cry16Aa1 (CAA63860) | Cry39Aa1 (BAB72016) | Cry64Aa1 (BAJ05397) |
| Cry17Aa1 (CAA67841) | Cry40Aa1 (BAB72018) | Cry65Aa1 (AEB52307) |
| Cry18Ba1 (AF169250) | Cry41Aa1 (BAD35157) | Cry66Aa1 (AEB52311) |
| Cry18Ca1 (AF169251) | Cry42Aa1 (BAD35166) | Cry68Aa1 (ADV33304) |
| Cry19Aa1 (CAA68875) | Cry43Ca (KC156676) | Cry70Aa1 (AEX56524) |
| Cry20Aa1 (AAB93476) | Cry43Cb (KC156695) | Cry72Aa1 (AGE10588) |
| Cry21Aa1 (I32932) | Cry44Aa1 (BAD08532) | Cry73Aa1 (AEH76822) |
| Cry22Aa1 (I34547) | Cry45Aa1 (BAD22577) | Cry74Aa1 (WP_029440438) |
| Cry23Aa1 (AF038048) | Cry46Aa1 (BAC79010) | Cry75Aa1 (ASY04853) |
| Cry24Aa1 (AAC61891) | Cry47Aa1 (AAY24695) |  |

**Table S4: Origin of toxin proteins used for searching *B. laterosporus* genomes**

| Protein name | Function | GenBank Accession No. | Organism | Reference |
| --- | --- | --- | --- | --- |
| Alv | Thiolactivated cytolysin | WP_003335622 | *Brevibacillus laterosporus* LMG 15441 | Djukic et al. 2011 |
| ETX/MTX2 | Etx/Mtx2 family beta pore forming toxin | WP_022584503.1 | *Brevibacillus laterosporus* strain PE36 |  |
| ETX/MTX2 | Etx/Mtx2 family beta pore forming toxin | WP_022584953.1 | *Brevibacillus laterosporus* strain PE36 |  |
| MTX4 | Etx/Mtx2 family beta pore forming toxin: | WP_080717387.1 | *Lysinibacillus sphaericus* | Berry 2012 |
|  | Peptidase | WP_022586504.1 | *Brevibacillus laterosporus* strain PE36 | Theodore et al. 2014 |
| Plx2B | Toxin 2B | AGJ74030.1 | *Paenibacillus larvae* | Funfhaus et al. 2013 |
| ChiA | Chitinase | AKN21157.1 | *Brevibacillus laterosporus* strain M64 | Liu and Miao unpubl. |
| ChiC | Chitinase | AKN79540.1 | *Brevibacillus laterosporus* strain M9 |  |
| Isp1a | Insecticidal secreted protein | CAI40767.1 | *Brevibacillus laterosporus* (strain not given) |  |
| Isp2a | Insecticidal secreted protein | CAI40768.1 | *Brevibacillus laterosporus* (strain not given) |  |
| Isp2b | Insecticidal secreted protein | WP_001996221.1 | *Bacillus cereus* |  |
| Vip1 | Vegetative insecticidal protein | AGC08395.1 | *Bacillus thuringiensis* | Bi et al. 2015 |
| Vip1A2 |  | AAR81088 |  | Feitelson et al. 2003” |
| Vip2B | Vegetative insecticidal protein | AGC08396.1 | *Bacillus thuringiensis* | Bi et al. 2015 |
| Vip2B |  | AAR40887 |  | Schnepf et al. 2003” |
| Vip2 | Vegetative insecticidal protein | 1QS1_A | *Bacillus cereus* |  |
|  | lethal factor domain protein | WP_099327290 | *Brevibacillus laterosporus* DSM25 |  |
| Vip4 | Vegetative insecticidal protein | AEE52299 | *Bacillus thuringiensis* |  |
| toxin2B |  | AGJ74030 | *Paenibacillus larvae* |  |
| Pebl1 | Protein elicitor | AJE60449.1 | *Brevibacillus laterosporus* strain A60 | Wang et al. 2015 |
| ExsC | cell wall proteins | AQX44452.1 | *Brevibacillus laterosporus* strain UNISS18 | Marche et al. 2017 |
| CHRD | cell wall proteins | AQX44453.1 | *Brevibacillus laterosporus* strain UNISS18 | Marche et al. 2017 |
| CpbA | cell wall proteins | AQX44450.1 | *Brevibacillus laterosporus* strain UNISS18 | Marche et al. 2017 |
| CpbB | cell wall proteins | AQX44451.1 | *Brevibacillus laterosporus* strain UNISS18 | Marche et al. 2017 |
| Cry18Aa | Pesticidal crystal protein | CCF16695.1 | *Brevibacillus laterosporus* strain GI9 | Sharma et al. 2012 |
| Cry27Aa | Pesticidal crystal protein | WP_016098322.1 | *Bacillus cereus* | Feldgarden et al. unpublished. |
| Cry35Aa1 | Insecticidal crystal protein | AAG50342.1 | *Bacillus thuringiensis* strain PS80JJ1 | Ellis et al. 2002 |
| BrvA | Brevibacillin synthetase A | ASV51722.1 | *Brevibacillus laterosporus* strain OSY-I1 |  |
| BrvB | Brevibacillin synthetase B | ASV51721.1 | *Brevibacillus laterosporus* strain OSY-I1 |  |
| BrvC | Brevibacillin synthetase C | ASV51723.1 | *Brevibacillus laterosporus* strain OSY-I1 |  |
| BrvD | Brevibacillin synthetase D | ASV51724.1 | *Brevibacillus laterosporus* strain OSY-I1 |  |
| BrvE | Brevibacillin synthetase E | ASV51725.1 | *Brevibacillus laterosporus* strain OSY-I1 |  |
| BrvF | Brevibacillin ABC transporter | ASV51726.1 | *Brevibacillus laterosporus* strain OSY-I1 |  |
| EcaA1 | calcium-transporting ATPase | XP_572412.1 | *Cryptococcus neoformans* var. *neoformans* JEC21 |  |
| PurL | Amidophosphoribosyl-transferase | NP_388531.2 | *Bacillus subtilis* subsp. *subtilis* str. 168 | Barbe et al. 2009 |
| Blg4 | alkaline serine protease | AAU81559.2 | *Brevibacillus laterosporus* G4 | Huang et al. 2005 |
| Enp | extracellular neutral proteases | ABI93802.1 | *Brevibacillus laterosporus* G4 | Tian et al. 2007 |

**Supplementary Figure S3.** Relationship between Vip and Isp type toxins used to interrogate genomes of *B. laterosporus.* Aligned proteins using MUSCLE, dendrogram generated in Fastree in Geneious. Lethal factor domain protein from *B. laterosporus* shows similarity to *B. anthracis* lethal factor in the N-terminal domain only (the region associated with interaction with protective antigen) and has a C-terminal ADP ribosyl transferase domain (in contrast to anthrax lethal factor, which has a metalloproteinase domain).

*
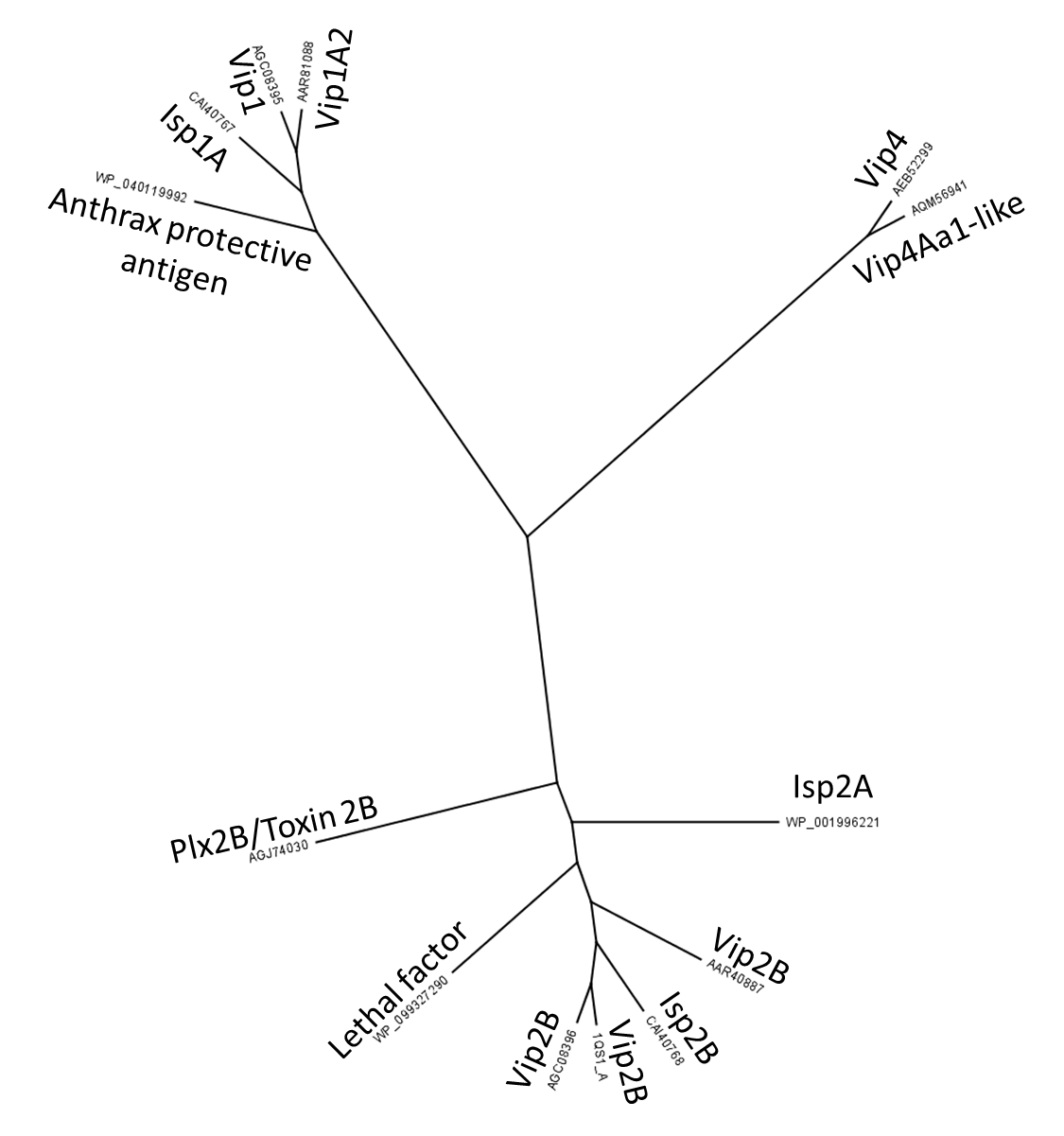
*

**Supplementary Table S5.** Distance matrix of comparison of Cry18-like proteins

|  | 1821L  cry18-like | B. laterosporus GI9  NZ_CAGD01000034  cry18Aa1 | 1821L  Cry18-like | B. laterosporus GI9  NZ_CAGD01000034 cry18Aa1 | *Paenibacillus popillae*  Cry18Aa1  CAA67506 | *Paenibacillus popillae*  Cry18Ba1  AF169250_2 | *Paenibacillus popillae*  Cry18Ca1  AF169251_1 |
| --- | --- | --- | --- | --- | --- | --- | --- |
| cry18Aa 1821 2 |  | 92.4 | 75.1 | 67.4 | 55.3 | 61.7 | 59.9 |
| NZ_CAGD01000034 crys - cry18Aa 2CDS translation |  |  | 74.8 | 65.9 | 58.9 | 61.8 | 61 |
| Cry18Aa 1821 1 |  |  |  | 79.6 | 54.5 | 59.3 | 58.7 |
| NZ_CAGD01000034 crys - cry18Aa1 CDS translation |  |  |  |  | 51.2 | 54.1 | 53.5 |
| CAA67506 |  |  |  |  |  | 62.5 | 59.7 |
| AF169250_2 |  |  |  |  |  |  | 72.4 |
| AF169251_1 |  |  |  |  |  |  |  |

**Supplementary Table S6**: Distance matrix of Vip and Isp like proteins (Figure S3) used for searching genomes. Percentage of residues which are identical between proteins.

|  | Isp2b  WP_001996221 | LF-like domain protein  WP_099327290 | Vip2B  AAR40887 | Isp2a  AI40768 | Vip2  1QS1_A | Vip2  AGC0839 | anthrax Protective antigen WP_040119992 | Isp1a  CAI40767 | Vip1  AGC08395 | Vip1Aa1  AAR81088 | Plx2B/  toxin2B  AGJ74030 | Vip4  AEB52299 | Vip4Aa  AQM56941 |
| --- | --- | --- | --- | --- | --- | --- | --- | --- | --- | --- | --- | --- | --- |
| WP_001996221 |  | 10.2 | 11.5 | 10.8 | 9.8 | 10.4 | 5.2 | 5.3 | 5.7 | 5.7 | 5 | 6 | 5.6 |
| WP_099327290 |  |  | 13.2 | 12.4 | 14.6 | 14.6 | 9.2 | 7.9 | 8.4 | 8.5 | 8.6 | 9.1 | 8.8 |
| AAR40887 |  |  |  | 60.6 | 69.4 | 68.9 | 6.7 | 9 | 9 | 8.3 | 8 | 7.9 | 9.2 |
| CAI40768 |  |  |  |  | 78 | 77.8 | 7.5 | 8.6 | 8.4 | 8.2 | 7.9 | 6.5 | 10.1 |
| 1QS1_A |  |  |  |  |  | 89.6 | 7.1 | 8.9 | 9.2 | 8.7 | 7.9 | 7.3 | 10.2 |
| AGC08396 |  |  |  |  |  |  | 7.1 | 8.8 | 8.8 | 8.5 | 8.1 | 6.9 | 10.2 |
| WP_040119992 |  |  |  |  |  |  |  | 24.1 | 24.2 | 23.9 | 24.3 | 21.5 | 30.3 |
| CAI40767 |  |  |  |  |  |  |  |  | 60.2 | 59.2 | 23.9 | 24.5 | 31 |
| AGC08395 |  |  |  |  |  |  |  |  |  | 74.3 | 26 | 25.7 | 31.2 |
| AAR81088 |  |  |  |  |  |  |  |  |  |  | 26.1 | 25.7 | 30.1 |
| AGJ74030 |  |  |  |  |  |  |  |  |  |  |  | 25.7 | 31.8 |
| AEB52299 |  |  |  |  |  |  |  |  |  |  |  |  | 37 |

**Supplementary Table S7:** BoNT cluster identities (5 identical residues)

**Region 1**

|  | **1821l** | **Rsp** | **1951** |
| --- | --- | --- | --- |
| **1821L** |  | 99.1 | 98.5 |
| **Rsp** | 99.1 |  | 99 |
| **1951** | 98.5 | 99 |  |

**Region 2 (associated with Cry27)**

|  | **1821L** | **Rsp** | **1951** |
| --- | --- | --- | --- |
| **1821L** |  | 97.8 | 96.7 |
| **Rsp** | 97.8 |  | 96.8 |
| **1951** | 96.7 | 96.8 |  |

**Supplementary Table S8: Distance matrix (% identity) of 7-8 Kb plasmids (generated using MUSCLE in Geneious)**

|  | Rsp | 1821L | CCEB342 | LMG15441 |
| --- | --- | --- | --- | --- |
| 1951 | 99.5 | 97.3 | 54.3 | 54.4 |
| Rsp |  | 96.9 | 54.2 | 54.2 |
| 1821L |  |  | 55 | 55 |
| CCEB342 |  |  |  | 100 |
| LMG15441 |  |  |  |  |

Additional references (only mentioned in the supplementary materials)

V. Barbe, S. Cruveiller, F. Kunst, P. Lenoble, G. Meurice, A. Sekowska, D. Vallenet, T. Wang, I. Moszer, C. Medigue and A. Danchin, From a consortium sequence to a unified sequence: the *Bacillus subtilis* 168 reference genome a decade later, Microbiol 155, 2009, 1758–1775.

Y. Bi, Y. Zhang, C. Shu, N. Crickmore, Q. Wang, L. Du, F. Song and J. Zhang, Genomic sequencing identifies novel *Bacillus thuringiensis* Vip1/Vip2 binary and Cry8 toxins that have high toxicity to Scarabaeoidea larvae, Appl Microbiol BiotechnolAppl. Microbiol. Biotechnol. 99, 2015, 753–760.

G. Delrio, D.J. Ellar, I. Floris, B. Paglietti, R.A. Pantaleoni, S. Rubino, L. Ruiu and A. Satta, 2011. *Brevibacillus laterosporus* strain compositions containing the same and method for the biological control of Diptera. U.S. Patent 8,076,119.

Feitelson, J.S., Schnepf, H.E., Narva, K.E., Stockhoff, B.A., Schmeits, J., Loewer, D., et al. (2003) Pesticidal toxins and nucleotide sequences which encode these toxins. US patent 6,656,908.

A. Funfhaus, L. Poppinga and E. Genersch, Identification and characterization of two novel toxins expressed by the lethal honey bee pathogen *Paenibacillus larvae*, the causative agent of American foulbrood, Environ MicrobiolEnviron. Microbiol. 15, 2013, 2951–2965.

Schnepf, H.E., Narva, K.E., Stockhoff, B.A., Lee, S.F., Walz, M. and Sturgis, B 2003. Pesticidal toxins and genes from *Bacillus laterosporus* strains. US patent 6,605,701
